# Evolution of NCoR-1 and NCoR-2 corepressor alternative mRNA splicing in placental mammals

**DOI:** 10.1186/s13104-019-4384-z

**Published:** 2019-06-17

**Authors:** Martin L. Privalsky, Michael L. Goodson

**Affiliations:** 1 Department of Microbiology and Molecular Genetics, College of Biological Sciences, University of California at Davis, Davis, CA 95616 USA; 2 Department of Anatomy, Physiology, and Cell Biology, College of Veterinary Medicine, University of California at Davis, Davis, CA 95616 USA

**Keywords:** Alternative mRNA splicing, Evolution, Corepressors, Transcriptional regulation, Nuclear receptors, Nuclear hormone receptors, NCoR, SMRT

## Abstract

**Objective:**

The NCoR-1 and NCoR-2 corepressors are products of an early gene duplication near the beginning of vertebrate evolution and play both overlapping and divergent roles in development and physiology. Alternative-splicing of NCoR-1 and NCoR-2 further customizes their functions. To better understand the evolutionary basis of this phenomenon we extended our prior study of NCoR-1 and NCoR-2 alternative-splicing to an expanded series of species.

**Results:**

Alternative-splicing of NCoR-2 was observed in all vertebrates examined whereas alternative-splicing of NCoR-1 was largely limited to placental mammals. Notably the most prominent of the NCoR-1 alternative-splicing events specific to the placental lineage, in exon 37 that plays a key role in murine metabolism, mimics in many features an analogous alternative-splicing event that appeared in NCoR-2 much earlier at the beginning of the vertebrate radiation. Detection of additional alternative-splicing events, at exons 28 in NCoR-1 or NCoR-2, was limited to the *Rodentia* or *Primates* examined, indicating both corepressor paralogs continued to acquire additional splice variations more recently and independently of one another. Our results suggest that the NCoR-1/NCoR-2 paralogs have been subject to a mix of shared and distinct selective pressures, resulting in the pattern of divergent and convergent alternative-splicing observed in extant species.

**Electronic supplementary material:**

The online version of this article (10.1186/s13104-019-4384-z) contains supplementary material, which is available to authorized users.

## Introduction

The NCoR-1 and NCoR-2 corepressors bind and mediate transcriptional repression by the nuclear receptor family of hormone-regulated transcription factors [1–5]. NCoR-1 and -2 share ~ 40% sequence identity and a common molecular architecture [5–7]. Both NCoR-1 and -2 possess N-terminal Silencing Domains (SDs) that recruit additional proteins to form a larger corepressor holocomplex, and C-terminal Receptor Interaction Domains (RIDs) that tether these holocomplexes to their nuclear receptor partners on target genes [6, 8–11]. Despite these commonalities NCoR-1 and -2 exert unique as well as overlapping molecular and biological functions [6, 8].

Whereas NCoR-1 and NCoR-2 are present as two distinct loci in all the craniate genomes analyzed, NCoR sequences in cephalochordates, urochordates, hemichordates, and echinoderms are encoded by a single locus [12]. NCoR-1 and -2 are therefore considered the products of gene duplication and divergence events that began near the origin of vertebrate evolution. Notably both NCoR-1 and -2 in mammals undergo alternative mRNA splicing that further broadens the diversity of actions available to each paralog and allows these actions to be customized for different purposes in different cell types [12–23], and in response to different signals [24, 25]. Understanding the evolution and extant patterns of utilization of these alternative-splicing events is important for understanding the biological roles of the different splice variants and how the functions of the NCoR paralogs have been customized to fit the differing requirements of different organisms.

We previously reported that whereas NCoR-2 alternative-splicing was detected in all the vertebrates examined, NCoR-1 splicing was apparently restricted to the placental mammals, an observation suggesting these two corepressors may have been subject to distinct forms of evolution subsequent to their gene duplication [23]. However our prior study was limited in scope with only 3 placental mammals and 5 non-placental vertebrates analyzed. To better understand the evolutionary relationships in this phenomenon, we have now expanded our analysis of alternative-splicing of NCoR-1 and NCoR-2 to a significantly wider series of eutherian lineages. Our new data help to confirm and elaborate on the concepts we proposed in our prior study, as well as providing a broader information base for further analysis and study.

## Main text

### Methods

**Fig. 1:**
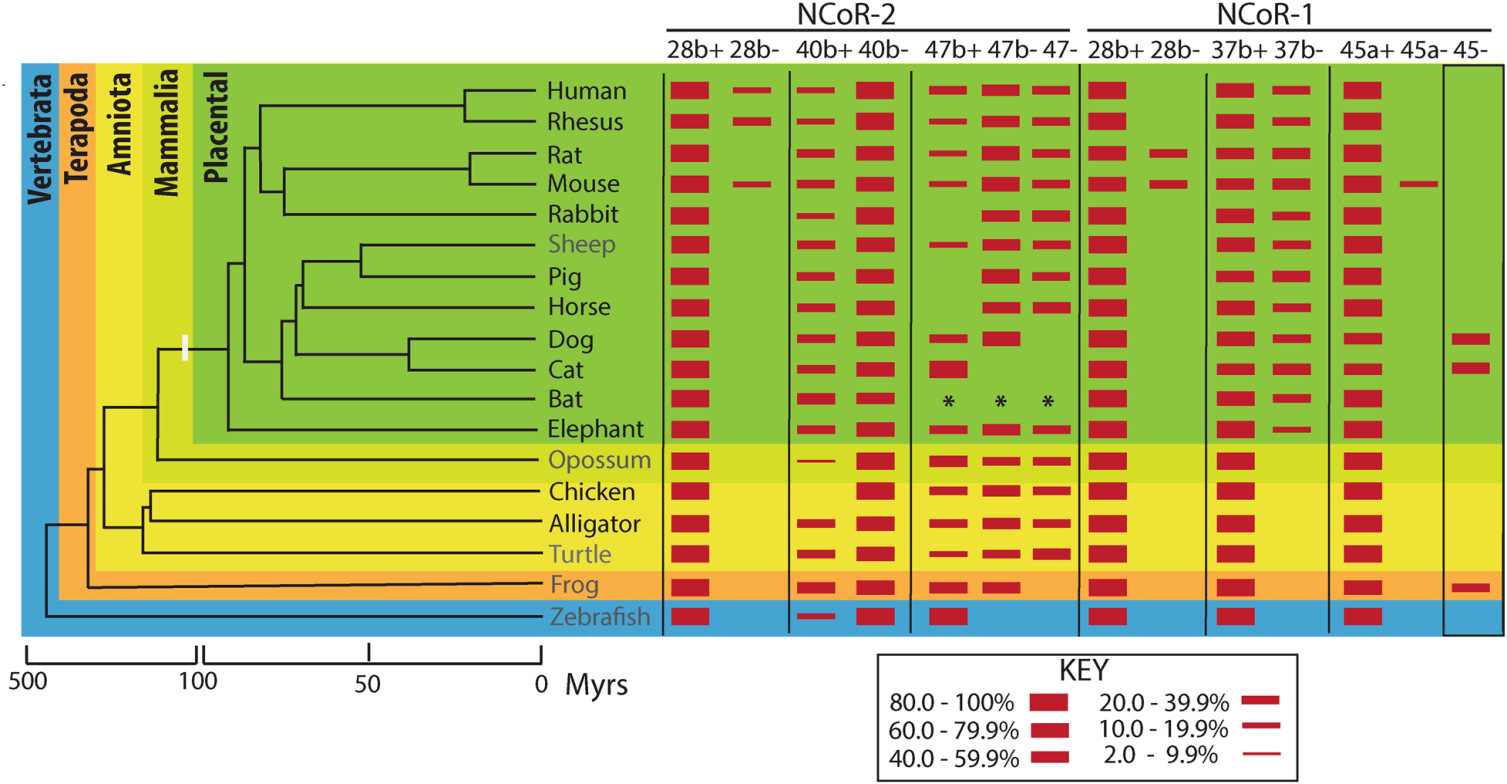
Schematic of alternative corepressor splicing compared to an evolutionary dendrogram. The abundance of each alternative-splice variant from each splice site analyzed is presented schematically as a red bar, with its thickness proportional to the percent of the total splice variants expressed from that site (= 100%, see key). Absence of a bar indicates “not detected” (< 2% of total expression at each splice site); see the Limitations section and Additional file 1 for an expanded discussion of the sensitivity and accuracy of the RT-PCR technique). The species/sources newly analyzed for the current study (Alligator, *Alligator mississippiensis*; Bat, *Eidolon helvum*; Cat, *Felis silvestris catus*; Chicken, *Gallus gallus domesticus*; Dog, *Canis lupus familiaris*; Elephant, *Loxodonta africana*; Horse, *Equus ferus caballus*, Human, *Homo sapien*; Mouse, *Mus musculus*; Pig, *Sus scrofa domesticus*; Rabbit, *Oryctolagus cuniculu*s; Rat, *Rattus norvegicus*; Rhesus, *Macaca mulatta*) are listed in black text and represent assays of RNA populations isolated from peripheral blood. Several additional species analyzed in a prior study [23], in grey text, are presented for comparison purposes and represent assays of RNA from non-hematological samples (Sheep, *Ovis aries* adult liver; Opossum, *Monodelphis domestica* adult liver; Turtle, *Trachemys scripta* adult liver; Frog, *Xenopus laevis* stage 45; Zebrafish, *Danio rerio* stage 84 h). The evolutionary relationships of the species are presented as a dendrogram with an estimate of divergence times (in millions of years, Mrys) presented below; please note break in time line. For ease of comparisons with other publications our prior nomenclature (NCoR and SMRT [23]) has been changed in the current manuscript to NCoR-1 and NCoR-2, respectively. The exon numbering system is that employed in our prior study [23]

Alternative-splicing was quantified from RNA prepared from nucleated peripheral blood and assayed by a reverse transcriptase PCR quantification method employing primers that spanned each alternatively-spliced site [14, 16, 17, 20, 21, 23, 25]. The primer sequences and a detailed description and discussion of the sensitivity and accuracy of this method are provided in Additional file 1. The relative abundance of each alternatively-spliced corepressor variant was calculated as a percentage of the sum of all the alternatively-spliced isoforms produced at that alternative-splice location. The mean and standard deviation of 3 or more experiments for each species were determined. Data from several species analyzed previously [23] are included in Fig. 1 for comparison. NCoR-2 was previously referred to as SMRT (an alternative nomenclature); the exon numbering system here is as in [23].

### Results

#### Convergent evolution at a nuclear receptor-specificity determinant within NCoR-1/2 exons 37/40

Alternative mRNA splicing at a receptor specificity determinant (RID-3) within exon 37 of mammalian NCoR-1 (mapping to exon 40 in NCoR-2) is known to have profound effects on the molecular and biological properties of these corepressors, including the control of lipid and glucose metabolism [13, 16–19]. Our new results confirm that although this alternative-splicing occurred in NCoR-2 in virtually all the vertebrates examined, alternative-splicing at this position in NCoR-1 was limited within our sensitivity of detection to the placental mammals analyzed (Table 1 and Fig. 1). Notably our new results also demonstrated that NCoR-1 exon 37 alternative-splicing occurred in all the placental mammals investigated, including *Proboscidea* (Table 1 and Fig. 1), indicative of a very early evolutionary acquisition of this trait within this clade and placing this convergent evolutionary event as arising in NCoR-1 300 to 400 million years after the appearance of the comparable alternative-splicing event in NCoR-2.

**Table 1:** Alternative splicing of NCoR-1 and NCoR-2 corepressors

Species	NCoR-1							NCoR-2						
	28b+	28b−	37b+	37b−	45a+	45a−	45−	28b+	28b−	40b+	40b−	47b+	47b−	47−
Alligator	100	ND	100	ND	100	ND	ND	100	ND	29.0 (4.4)	71.0 (4.4)	22.4 (5.3)	40.0 (6.0)	37.6 (5.3)
Bat	100	ND	73.1 (8.7)	26.9 (8.7)	100	ND	ND	100	ND	52.5 (9.0)	47.5 (9.0)	*	*	*
Cat	100	ND	59.8 (1.7)	40.2 (1.7)	49.0 (2.3)	ND	51.0 (2.3)	100	ND	25.1 (1.2)	74.9 (1.2)	100	ND	ND
Chicken	100	ND	100	ND	100	ND	ND	100	ND	ND	100	24.6 (3.8)	41.8 (3.9)	33.6 (3.6)
Dog	100	ND	64.4 (3.9)	35.6 (3.9)	59.5 (2.2)	ND	40.5 (2.2)	100	ND	21.7 (2.6)	78.3 (2.6)	28.0 (4.5)	72.0 (4.5)	ND
Elephant	100	ND	89.4 (3.3)	10.6 (3.3)	100	ND	ND	100	ND	30.4 (2.9)	69.6 (2.9)	24.3 (3.1)	46.0 (5.3)	29.7 (5.5)
Horse	100	ND	69.3 (6.8)	30.7 (6.8)	100	ND	ND	100	ND	26.8 (5.4)	73.2 (5.4)	ND	46.4 (6.6)	53.6 (6.6)
Human	100	ND	70.2 (5.5)	29.8 (5.5)	100	ND	ND	86.0 (2.5)	14.0 (2.5)	17.5 (3.9)	82.5 (3.9)	20.4 (1.8)	53.6 (1.5)	26.0 (1.9)
Mouse	76.2 (0.2)	23.8 (0.2)	54.2 (2.4)	45.8 (2.4)	88.8 (0.9)	11.2 (0.9)	ND	86.9 (2.7)	13.1 (2.7)	32.9 (2.3)	67.1 (2.3)	17.2 (1.8)	61.2 (4.2)	21.6 (2.8)
Pig	100	ND	55.0 (1.5)	45.0 (1.5)	100	ND	ND	100	ND	27.9 (0.6)	72.1 (0.6)	ND	64.5 (1.7)	35.5 (1.7)
Rabbit	100	ND	63.8 (1.3)	36.2 (1.3)	100	ND	ND	100	ND	15.2 (1.1)	84.8 (1.1)	ND	54.4 (3.4)	45.6 (3.4)
Rat	73.2 (1.7)	26.8 (1.7)	58.0 (3.6)	42.0 (3.6)	100	ND	ND	100	ND	32.8 (2.0)	67.2 (2.0)	17.3 (2.1)	60.3 (1.6)	22.3 (3.1)
Rhesus	100	ND	63.8 (5.4)	36.2 (5.4)	100	ND	ND	69.7 (2.6)	30.2 (2.6)	19.5 (3.0)	80.5 (3.0)	19.5 (2.1)	58.6 (6.1)	22.0 (6.4)

Alternative mRNA splicing was determined as described in "Methods" and Additional file 1 from RNA isolated from peripheral blood from each species listed (Alligator, *Alligator mississippiensis*; Bat, *Eidolon helvum*; Cat, *Felis silvestris catus*; Chicken, *Gallus gallus domesticus*; Dog, *Canis lupus familiaris*; Elephant, *Loxodonta africana*; Horse, *Equus ferus caballus*, Human, *Homo sapien*; Mouse, *Mus musculus*; Pig, *Sus scrofa domesticus*; Rabbit, *Oryctolagus cuniculus*; Rat, *Rattus norvegicus*; Rhesus, *Macaca mulatta*). Results from an individual of each species are presented. The relative abundance of each alternative-splice variant from each splice site analyzed is presented as a percent of the total splice variants expressed from that site (sum = 100%)

ND = not detected (limits of detection were estimated as < 2% of total expression at each splice site, the limits of accurate quantitation as > 5%). The structures of the alternative-spliced transcripts are detailed in [23] except for the NCoR-1 exon 45− splice variant, which was newly recognized from the current analysis and was confirmed by sequence analysis of the PCR products. The mean and (standard error) of 3 or more analyzes are shown for each value

* Data not available. See “Limitations” and Additional file 1 for an expanded discussion of the sensitivity and accuracy of this RT-PCR methodology

#### Divergent evolution at a nuclear receptor-specificity determinant within NCoR-2 exon 47

Our current results also documented that alternative-splicing at a second site important for nuclear receptor-specificity, within NCoR-2 exon 47 and encompassing/flanking RID-1 [15], was widely distributed among all the vertebrate lineages examined, but in contrast to the exon 37/40 splice no comparable alternative-splice form was detected in the NCoR-1 paralog in any species tested (Table 1 and Fig. 1). We interpret this alternative-splicing event as a divergent acquisition by the NCoR-2 corepressor early after duplication of the common ancestral gene that, unlike exon 37/40 alternative-splicing, retained a paralog-restricted role over subsequent evolutionary time.

#### Alternative-splicing events at positions likely to influence functions of the corepressor other than nuclear receptor specificity

Additional alternative-splicing events were observed in our analysis at NCoR-1 and NCoR-2 exons 28 that were apparently restricted to the *Primates* and *Rodentia* examined and were probably acquired relatively late in placental mammalian evolution (Table 1 and Fig. 1). These mapped to or near corepressor regions, such as SD sequences, that are likely to influence assembly or other functions of the corepressor complex rather than nuclear receptor specificity. The precise locations and natures of these alternative-splice sites are non-identical in NCoR-1 versus NCoR-2, consistent with these alternative-spliced also representing the products of divergent evolutionary events.

#### Newly recognized, taxonomically-restricted alternative-splicing at the C-terminus of NCoR-2

The current study also newly detected an alternative-splice variant of NCoR-1 that deletes all of exon 45 (exon 45-, Table 1 and Fig. 1), removing portions of a corepressor domain associated with Mitogen-Activated Kinase cascade-regulation [24]. This NCoR-1 splice variant was not seen in NCoR-2 and was only detected in the two *Carnivora* tested and in an *Anura*. The latter is notable in that it represents the only observed example of any form of NCoR-1 alternative-splicing in non-mammals. Our failure to previously detect this splice form was a consequence of the unanticipated small size of the corresponding PCR product generated by the primers in our prior studies.

### Discussion

Both NCoR-1 and -2 are subject to alternative mRNA splicing, generating an extensive series of corepressor protein variants that are expressed at different abundances in different tissues, preferentially associate with different transcription factor partners, respond to signal transduction pathways in distinct ways, and play distinguishable, even opposing, biological roles [13, 16–20]. This alternative-splicing is a major determinant of NCoR corepressor function that further broadens the diversity of actions available to each paralog and allows these actions to be customized for different purposes in different cell types and in response to different signals. Understanding and comparing the evolution and extant patterns of utilization of these alternative-splicing events in NCoR-1 versus NCoR-2 should therefore provide important insights into the biological roles of the different splice variants and how the functions of these paralogs have been customized to fit the differing requirements of different organisms.

With this goal we report here an expanded analysis of alternative-splicing in NCoR-1 and NCoR-2 to more fully understand the origins of this phenomenon. Our studies revealed that there are both commonalities and substantial differences in the alternative splicing of NCoR-1 versus NCoR-2, presumably reflecting and helping to mediate the multiple biological roles of these two paralogs.

We previously suggested, based on analysis of a limited number of species, that NCoR-2 alternative-splicing likely occurs in all vertebrates, whereas NCoR-1 splicing is restricted to the placental mammals [23]. Our current expanded study strengthens that hypothesis (with one exception, see below) and given the very early divergence of *Afrotheria/Proboscidea* during the placental mammal radiation, our new data places the acquisition of NCoR-1 alternative-splicing as likely having occurred during the Cretaceous period some 300 to 400 million years after the acquisition of alternative-splicing by NCoR-2. Further, despite its later evolutionary appearance, the most prominent and wide spread of the alternative-splicing events in mammalian NCoR-1, at the RID-3 domain in exon 37, mimics in many of its structural and molecular features a RID-3/exon 40 alternative-splicing event acquired by NCoR-2 at the beginning of the vertebrate radiation. Alternative-splicing at these sites alters the affinity of the corepressor proteins for their nuclear receptor partners, including those important in control of lipid and carbohydrate metabolism. We speculate that the later acquisition of this alternative-splicing event by NCoR-1 in the placental mammals may reflect a unique role for this corepressor variant in energy homeostasis in this lineage.

Additional alternative-splicing events, at NCoR-1 exon 28 and 45, and at NCoR-2 exon 28 and 47, were each unique to their corresponding paralog and appear to be divergent evolutionary events. The NCoR-2 exon 47 alternative-splice site maps to RID-1 and is known to alter the affinity of the encoded corepressor protein toward a subset of its nuclear receptor partners. In contrast the NCoR-1 exon 28 and NCoR-2 exon 28 splicing events map not to RIDs but to regions likely to influence assembly (SD domains) or other functions of the corepressor complex. These exon 28 splicing events were non-identical in NCoR-1 and NCoR-2 and their detection was limited to the *Rodentia* and *Primates* examined, suggesting that both paralogs continued to acquire additional splice variations within the past 90 to 100 million years and did so independently of one another.

Our new analysis also revealed a previously unrecognized alternative-splice event in NCoR-1 exon 45 that removed all of exon 45 and was found exclusively in the two *Carnivora* tested and in the amphibian *Xenopus*. The only other alternative-splice event known at this exon removes only a portion of exon 45 [exon 45a−] and is limited to mice. The basis for this tight species restriction is unclear, as are the exact effects of these splicing events on NCoR-1 function, although based on location they may alter regulation of NCoR function by MAP kinase cascade signaling. The presence of the exon 45− splice in *Xenopus* is the first known example of NCoR-1 alternative-splicing in a non-mammal. Analysis of additional amphibian and related species will be required to better understand the taxonomic extent, mechanistic origin, and biological implications of this unanticipated observation.

In conclusion our results are most consistent with the NCoR-1 and NCoR-2 corepressor paralogs having been subject to a mix of shared and distinct selective pressures that differed in different lineages, leading to the current pattern of divergent and convergent alternative-splicing events observed in extant species.

## Limitations

This study focuses on alternative-spliced sites within NCoR-1 and -2 that were previously recognized and in many cases established as having molecular or biological effects; there may be additional sites of alternative-splicing in one or more species not queried by the primer pairs used here. Ethical and access limitations in working with many of the species required the current study to be restricted to analysis of peripheral blood samples; we cannot fully exclude the possibility that a splice variant not detected in peripheral blood may nonetheless be expressed by that species in another tissue. Nonetheless our prior studies on model organisms suggest that the detected presence or absence of a given variant in peripheral blood is largely predictive of its detected presence or absence in other tissues in that organism (e.g. [23]). Similarly although our methodology can detect alternative-splice products at levels down to ~ 2% of total we cannot exclude certain splice variants were not observed because they are expressed at levels even below this limit; such very low levels of expression, if they exist, would be of unclear biological significance.

Due to practical and ethical limitations it was typically not possible to obtain samples from multiple individuals from within a given (exotic) species; however our analysis of model organisms such as human and mice demonstrated relatively little variation in alternative-splicing from individual to individual (e.g. [14, 16, 17, 20, 23, 25] and data not shown), indicating that this restriction was unlikely to substantially alter the interpretation of the data presented here. Finally our methodology required access to freshly drawn peripheral blood and sufficient species-specific sequence information to design appropriate PCR primers. These dual requirements restricted our ability to expand our analysis to taxonomies not reported here or to include additional representatives within the lineages that were studied. Nonetheless the patterns of alternative mRNA splicing were generally very similar in the cases where we were able to compare two species within a shared lineage.

## Additional file

**Additional file 1.** Expanded methods. This file details the RT-PCR methodology, the oligonucleotide primers employed, and the issues relating to the quantitation and sensitivity of the data.

## Abbreviations

NCoR: nuclear corepressor
RID: receptor interaction domain
SD: silencing domain

## Availability of data materials

All data generated or analyzed during this study are included in this published article.
