# Supplementary material for: Evolution of NCoR-1 and NCoR-2 corepressor alternative mRNA splicing in placental mammals
Source: BMC Res Notes. 2019 Jun 17;12:343. doi: 10.1186/s13104-019-4384-z (PMC6580476; doi:10.1186/s13104-019-4384-z)
Supplement: Supplementary file 1 — Additional file 1. Expanded methods. This file details the RT-PCR methodology, the oligonucleotide primers employed, and the issues relating to the quantitation and sensitivity of the data. [file 13104_2019_4384_MOESM1_ESM.docx]

**ADDITIONAL FILE 1: Expanded Methods.**

Isolation of RNA, cDNA synthesis, and PCR analysis. RNAs were prepared from nucleated peripheral blood using a LeukoLock Total RNA Isolation System (ThermoFisher, Waltham MA) and the manufacturer’s protocol. Approximately 1 μg of total RNA from each sample was converted into cDNA using a QuantiTect Reverse Transcription Kit (Qiagen, Hilden, Germany) following the manufacturer’s directions and including a DNA-wipeout pre-step to avoid genomic contamination. Two μl of each 120 μl cDNA preparation were amplified by polymerase chain reaction (PCR) in a total 20 μl volume using oligonucleotide primer pairs flanking each alternative splice-site (Supplemental Table S1 below), GoTaq enzyme, GoTaq buffer (Promega, Madison WI), and following the manufacturer’s recommendations. PCR was typically performed at 94 ^o^C for 2 mins. followed by 30 cycles of 30 secs. at 94 ^o^C, 30 secs. at 53 ^o^C, 60 secs. at 72 ^o^C, and a final 5 min. extension at 72 ^o^C.

The PCR products from each primer pair were resolved by electrophoresis in a 2% agarose gel in 1X TAE buffer, and the DNA was stained with ethidium bromide and quantified by use of a digital camera and AlphaEase software [1-3]. The abundance of each alternatively-spliced corepressor isoform was calculated as a percentage of the sum of all the alternatively-spliced isoforms produced at that alternative-splice location (primers were designed so that the different isoforms derived from a given splice site yielded distinct PCR products [1-3].

Each sample was analyzed by PCR a minimum of 3 times and the average and standard error calculated. Analysis of the species presented here was restricted to samples from single individuals (reflecting practical/ethical considerations); however only minimal variations were observed when different individuals from a single species were analyzed, with the variation between individuals comparable to the variation when a sample from one individual was analyzed multiple times (e.g. [2] and data not shown).

Accuracy, limitations, and interpretation of the RT-PCR methodology. Although considered poor at quantitation of absolute nucleic acid levels, the end-point RT-PCR technique as employed here is effective at measuring relative abundances, allowing the percentages of the different splice variants produced at each alternative-splice site to be accurately determined (e.g. [1-6]). Specifically:

The RT-PCR methodology as employed represents a highly internally-controlled technique: all isoforms from a given splice site are amplified in parallel in the same tube at the same time under identical conditions using the same up and down primers. The method is therefore relatively insensitive to non-uniformities in primer efficiency, reaction assembly, pipetting, temperature, ramp-rate, lane-loading, electrophoresis, staining or destaining.

To further minimize potential differences in the efficiency of amplification of the different isoforms our primers were designed to have comparable hybridization temperatures and to minimize the size differences between the PCR products produced from each splice site as much as possible while still allowing the different isoforms to be distinguished. The primers were also designed so that the PCR products derived from different species were close in size to one another to ensure similar efficiencies of amplification of the same isoform throughout all the species examined. We also limited the maximum size of our PCR products to below ~1000 bases and employed an extension time (60 seconds) calculated to allow completion of both the longest and shortest PCR products at each cycle (e.g. [7]).

The reproducibility of this method has been confirmed in practice by the low standard deviations observed when this approach was repeated at distinct times or by different researchers (e.g. [3]). The relative ratios of the PCR products generated from a given splice site were not significantly altered over a wide range of cycle numbers or input cDNA concentrations (Supplemental Tables S2 and S3 below).

Comparison of the end-point RT-PCR method to alternative approaches. Application of a qRT-PCR protocol would require primers able to selectively amplify the isoforms that lack a given exon but not the corresponding isoforms that contain that exon (as well as *vis-versa*). One potential solution to this conundrum would be creation of primers (or Taqman probes) that span each alternative exon such that their complementary sequences are intact in the exon (-) species but are separated in the exon (+) isoform. Successful design and use of such spanning oligonucleotides depend heavily on their ability to differentially hybridize to the exon (-) and (+) forms, which in practice is difficult to achieve with high specificity and consistent amplification efficiency for the multiple splice forms/species under study. RNAseq is another approach to quantification of the different splice forms, but is both costly and highly effort intensive given the number of species under investigation and the depth of the paired-end reads necessary for generating the statistics needed to detect and quantitate the low abundance isoforms. Taking these concerns under consideration, and to preserve consistency with prior published results, we chose to employ the end-point RT-PCR methodology as described for the current study.

References cited

**1.** Goodson ML, Mengeling BJ, Jonas BA, et al. Alternative mRNA splicing of corepressors generates variants that play opposing roles in adipocyte differentiation. J Biol Chem*.* 2011;286(52):44988-44999.

**2.** Privalsky ML, Snyder CA, Goodson ML. Corepressor diversification by alternative mRNA splicing is species specific. BMC Evol Biol*.* 2016;16(1):221-234.

**3.** Snyder CA, Goodson ML, Schroeder AC, et al. Regulation of corepressor alternative mRNA splicing by hormonal and metabolic signaling. Mol Cell Endocrinol*.* 2015;413:228-235.

**4.** Malartre M, Short S, Sharpe C. Alternative splicing generates multiple SMRT transcripts encoding conserved repressor domains linked to variable transcription factor interaction domains. Nucleic Acids Res*.* 2004;32(15):4676-4686.

**5.** Mengeling BJ, Phan TQ, Goodson ML, et al. Aberrant corepressor interactions implicated in PML-RAR(alpha) and PLZF-RAR(alpha) leukemogenesis reflect an altered recruitment and release of specific NCoR and SMRT splice variants. J Biol Chem*.* 2011;286(6):4236-4247.

**6.** Short S, Malartre M, Sharpe C. SMRT has tissue-specific isoform profiles that include a form containing one CoRNR box. Biochem Biophys Res Commun*.* 2005;334(3):845-852.

**7.** Montgomery JL, Rejali N, Wittwer CT. The influence of nucleotide sequence and temperature on the activity of thermostable DNA polymerases. J Mol Diagn*.* 2014;16(3):305-313.

**Supplemental Table S1. Primer pairs employed in the RT-PCR analysis**

| **Species** | **NCoR-1** | | | **NCoR-2** | | |
| --- | --- | --- | --- | --- | --- | --- |
|  | Exon 28 | Exon 37 | Exon 45 | Exon 28 | Exon 40 | Exon 47 |
| Alligator | ctgtctcagtctggcattgc  ggctttcccttggtaatggc | ccagctacgtatcatgcagc  gtggcctgtaccgatgaaac | ggggaggatcatggagttgt  gatggatgttggcggtgtac | ggcacccgatcccattaaac atgtgatgggcgattctgga | cgtatagcagaggacagggg  agtcatcgagggcttcagag | acccgggacagagattttca  gtactgtgagcagagcaggg |
| Bat | tggtcagggcacaacatgaa  tgctcgtatgggaggactct  ggcacaacatgaaggagtgg  gaaccagacagcacagtcct | ggcccttcaataagccaagg  aggcctgtagtttctcctgg  tctgctccccagatggatgt  ggtgaactggcagggcttat | agcttgaaaacacgtcaccc  taaggaaactgagttgagcc  agcttgaaaacacgtcaccc  tgttctgttggtgaggagct  agcttgaaaacacgtcaccc  ggctctcgctcccagatcct | cccaccatctccaaccctcc  cccttcttgccctcgtaga  ctcatctcctccaccaagca  cccttcttgccctcgtaga | ggacctgcagcgagaaaaga  tagtcctgcgtgatgacctc  cctgcagcgagaaaagactc  tagtcctgcgtgatgacctc | No data obtained. |
| Cat | ctctgtcacaggctggcata  tcttcaaagctttcggtggt | cccttcagtgagagctcagg  ggtctgctggcttcactttc | gagtcgtcatgtcccaacct  ccgcatagtcagagggttgt | ccacgtcccttactcagagc  ccttcttgccctcgtagatg | caccgggaaaagactcaaag  accaccctctggtgaccttt | gacggagatcttcaacatgc  catgatcagggggttgtagg |
| Chicken | cgagaggaagcttcagccaa  ggctccttcgaatgccctta | cttcccgctacaatacggct  tctgcctggcttggtttagg | gacgacaagagcgaggatca  tactgctgagcatccgcatt | gccactgacaatggatccca  tcactttctccgctctgctg | ggagtcgaggcctttacctg  ccctttctcgtgggataggc | gcagcctgggacagagattt  cccctctgagtgcactgaag |
| Dog | gaggaagctgcatcaaaagg  tgctcgtatgggaggactct | tgctcagctacgaatcatgc  gctggcttcactttctgctt | tgccaacacctcagtggtaa  agcatccgcatagtcagagg | atgtcccatactccgagcac  cctggtgatggtgcctttat | gcatcgggaaaagactcaaa  ccctccaggtgactcttgtc | cttcaacatgcctgccatc  agggggttgtaaggaaatgg |
| Elephant | agagaggaagctgcatccaa  gccccatttctttgatggta | gctcagaacagcctggtagg  caggtgaactggcaggactt  agcctcccgttacaacactg  tcggtagtggtgcaatggtc | cagtggagagacacgcagag  actatccgacagcgtctcgt | cgtgtctgttcagctccatg  gcccttcttgccctcataga | ttccatccaggaattggaac  ggtggtgccgtgtgtagtc | acacccatagccggaatgac  gacttggctttccgactgct  caactccgccatggtcagat  gacttggctttccgactgct  caactccgccatggtcagat  tccgagtgcacagatgagac  acacccatagccggaatgac  tccgagtgcacagatgagac |

| Horse | actgaggctttggtgaaggg  gttgttgctcttggtgtgcc | cctcccgttacaacactgct  ttggtttgccttcaccacct | cggagttgtcctgtcccaaa  caggctctcgttcccagatc | cccctcatctcctcctcctccaa  tggtgatggtgcccttgtac | tgcaccgggaaaagactcaa  ctggatgaaggctggctctc | cggggacagagatcttcaat  ccgcatgatcagtgggttgt  cttcaatatgcctgccatca  ccgcatgatcagtgggttgt |
| --- | --- | --- | --- | --- | --- | --- |
| Human | aggctttggtgaaggggtcca  tcagttgttgctcttggtgtc | acagagacccagtgttttccaag  gcaggacttatcacctcaatagca | ccagtaatcttgggctggaa  ccgacagggtctcgtactgt | aatgtcggtccagctccacgt  cctttggtgatgcttccgcc  tgccatctcccaaggaatgt  gtgatggtgcccttgtacag | cggacccgcaccgggaaaagac  aggtgcttggggagccccttg | caagaagctgaacacccaca  cgagtgcactgaggagacag |
| Mouse | gccatgttatctatgaaggcaaaa  agtcctctctttgaggtcagaatg | ctggctgctcttgtggatgc  ctgtcccattccctctgactg | ttcttttgctgatcccgcca  gcatccgtatggtcagaggg | gatgtcagtccagcttcgtgtg cccttggtgatgcttccact | ccatccaggaattggaactccg  tcaccgggctgatgggctc | aacaagaaactcaacacccacac  ctgcctgtagcctcataatcaaag |
| Pig | ggcgttggtaaagggatcca  acttgaggccgtcttcgaag | gtgatcacccctttggaccc  aactggcagggcttatcacc | ttaacctgccagctgtcaa  cagatcctgctctgctggtg  cttcgacgacaaggtggag  ggttgtaggggaactgggtg  cttcgacgacaaggtggag  cagatcctgctctgctggtg | catctcccagggaatgtcgg  ccctcgtagatgacatggcc | cggacctacaccgggaaaag  ctggtgacctttgacccctg | cagcctgggacggaaatctt  ggggttgtaggggaaaggtg |
| Rabbit | gaagaagctgcatccaaagg  tccggctctcctgagttaaa | ggacccaactgctcagctac  aggtgagctggcaggactta | ctctcgaggccattcttttg  gggggttataagggaactgc | ctcatctcctccaccaagca  agtgatggtgcccttgtaca | gcaccgggaaaagactcag  gtggctcttgtcgagctctt | cttcaatatgcccgccatc  gcatgatcagggggttgtag |

| Rat | gccatgtcatctatgaaggcaaaa  acgccccatttctttgatgg | ctgcaccccagatggatgtt  ctgtcccattccctctgcctg | ttcttttgctgatcctgcc  gcatccggatggtcagaggg | gaatgtcagtgcagcttcgcgtg  cgatcctgctgatggtaccc  gaatgtcagtgcagcttcgcgtg  cctgctgatggtacccttgt  tgtcagtgcagcttcgcgtg  cgatcctgctgatggtaccc  tgtcagtgcagcttcgcgtg  cctgctgatggtacccttgt | ccatccaggaattggaactccg  ccctctggtgacctttgatg | cagcctgggacggaaatctt  ctgcctgtagcctcataatcaaag |
| --- | --- | --- | --- | --- | --- | --- |
| Rhesus | agagaggaagctgcatccaa  tgccatcgtatggttttcct | ttccaaggaaccaatggaac  catcgttttcggcttgattt | aaggctctcatgggaagctt  tgttctgttggtgaggagct | tgccatctcccaaggaatgt  tgcccttgtacaggacatca  aatgtcggtccagctccacgt  cctttggtgatgcttccgcc | cggacccgcaccgggaaaagac  aggtgcttggggagccccttg | tcttcaatatgcccgccatc  ctgagtgcactgaggagac |

The primer pairs employed in the rtPCR assay are listed. Each pair spans the alternatively-spliced site noted, with the upper sequence of each pair representing the upstream primer (highlighted in green) and the lower sequence of each pair representing the downstream primer (highlighted in blue). Primers were designed such that each splice variant yielded a distinct-sized PCR product. More than one primer pair was employed at several splice sites for purposes of further confirmation or extended quantitation.

**Supplemental Table S2. Effect of Cycle Number on End Point RT-PCR Quantitation**

| **Cycle Number** | **NCoR-1**  37b+ 37b**-** | | **NCoR-2**  40b+ 40b- | | **NCoR-2**  47b+ 47b- 47- | | |
| --- | --- | --- | --- | --- | --- | --- | --- |
| **22** | 79.1% | 20.9% | * | * | * | * | * |
| **24** | 77.4% | 22.6% | 18,5% | 81.5% | 14.8% | 70.0% | 15.2% |
| **26** | 80.1% | 19.9% | 14.9% | 85.1% | 9.2% | 81.4% | 9.4% |
| **28** | 82.8% | 17.2% | 16.1% | 83.9% | 10.1% | 80.7% | 9.1% |
| **30** | 82.0% | 18.0% | 14.2% | 85.8% | 10.5% | 78.5% | 10.9% |
| **32** | 81.7% | 18.3% | 13.0% | 87.0% | 11.7% | 71.9% | 16.4% |
| **34** | 77.6% | 22.4% | 17.4% | 82.6% | 13.9% | 71.0% | 15.1% |

The overall RT-PCR protocol was as described for Table 1 of text. Two microliters of human liver cDNA were employed as input per reaction and the cycle numbers are indicated. *Too low a yield to quantitate the relative percentages accurately.

**Supplemental Table S3. Effect of DNA Input on End Point RT-PCR Quantitation**

| **DNA Input** | **NCoR-1**  37b+ 37b**-** | | **NCoR-2**  40b+ 40b- | | **NCoR-2**  47b+ 47b- 47- | | |
| --- | --- | --- | --- | --- | --- | --- | --- |
| **0.031 μl** | 78.0% | 22.0% | 18.3% | 81.7% | * | * | * |
| **0.062 μl** | 77.4% | 22.6% | 19.9% | 80.1% | * | * | * |
| **0.125 μl** | 83.9% | 16.1% | 16.3% | 83.7% | 5.8% | 75.1% | 8.2% |
| **0.25 μl** | 80.5% | 19.5% | 14.1% | 85.9% | 7.1% | 85.5% | 7.5% |
| **0.5 μl** | 76.7% | 23.3% | 14.5% | 85.5% | 6.2% | 86.1% | 7.7% |
| **1.0 μl** | 75.9% | 24.1% | 12.5% | 87.5% | 6.3% | 84.2% | 9.3% |
| **2.0 μl** | 77.2% | 22.8% | 13.0% | 87.0% | 7.4% | 83.0% | 9.6% |

The overall RT-PCR protocol was as described for Table 1 of text. Thirty-two PCR cycles were employed for all samples and the input DNA levels per reaction are indicated (1.24 μg nucleic acid/μl from human liver). *Too low a yield to quantitate the relative percentages accurately.
